# Evaluation of the effects of a digital health platform on business and medical practices of informal medicine vendors in Lagos, Nigeria

**DOI:** 10.1093/oodh/oqae035

**Published:** 2024-12-02

**Authors:** Laura A Ruiz-Gaona, Jed Friedman, Nejma Cheikh, Thomas Wilkinson, Mengxiao Wang, Jasmine Vicencio, Sohail Agha, Marelize Gorgens

**Affiliations:** Health Sector team, Abdul Latif Jameel Poverty Action Lab (J-PAL), 400 Main Street, Cambridge, MA 02142, USA; Development Research Group, World Bank, 1818 H Street NW, Washington DC 20433, USA; Health and Nutrition Global Practice, World Bank, 1818 H Street NW, Washington DC 20433, USA; Health and Nutrition Global Practice, World Bank, 1818 H Street NW, Washington DC 20433, USA; Health and Nutrition Global Practice, World Bank, 1818 H Street NW, Washington DC 20433, USA; Health and Nutrition Global Practice, World Bank, 1818 H Street NW, Washington DC 20433, USA; Global Health Visions, Saugerties, 186 Hommelville Rd, NY 12477, USA; Health and Nutrition Global Practice, World Bank, 1818 H Street NW, Washington DC 20433, USA

**Keywords:** digital health platform, business practice, impact evaluation, Lagos, NaijaCare, Nigeria, PPMV

## Abstract

An online platform—entitled NaijaCare—offered digital ordering, business development, peer exchange and business skills training to informal medicine vendors in Lagos, Nigeria. Outcomes of these vendors (known as PPMVs—patent and proprietary medicine vendors) who had participated in an earlier round of NaijaCare programming are compared with those of other PPMVs operating in the same local markets. Program impacts are estimated using a difference-in-differences estimator, with inverse propensity weighting to balance possible differences in baseline characteristics between intervention and control PPMVs. NaijaCare’s full range of features did not lead to significant improvements in most of the main outcomes of interest including: a business practice index (−1.4 ± 1.6, *P =* 0.09), the number of non-counterfeit medicine provided (0.2 ± 0.4, *P =* 0.25), client perception of PPMV quality (−0.4 ± 0.3, *P =* 0.03), changes in number of regular clients (−4.0 ± 5.3, *P =* 0.13) and PPMV role during the COVID-19 pandemic (e.g. not significantly more likely to sell a range of COVID prevention products). Among all participating PPMVs, engagement with the platform was low thereby highlighting engagement as a key determinant of platform success. The subset of PPMVs that did frequently use NaijaCare not only improved their record-keeping practices (0.28 ± 0.27, *P =* 0.05) but also reported significantly lower daily profits than control PPMVs (−3759 ± 3417, *P =* 0.04), possibly indicating that push factors for platform engagement, such as business hardship, were responsible for observed adverse outcomes. As platform availability coincided with a tumultuous period of change brought on by the COVID-19 pandemic, these disruptions may have limited intervention use and effectiveness.

**RESUMEN:**

Una plataforma en línea – llamada NaijaCare – ofreció servicios digitales de pedidos, desarrollo empresarial, intercambio entre iguales, y formación en habilidades comerciales a vendedores informales de medicinas en Lagos, Nigeria. Los resultados de vendedores (conocidos como PPMVs por las siglas en inglés de ‘vendedores de medicinas de marca y patente’) que participaron en una ronda de uso de los programas de NaijaCare se comparan con los de otros PPMVs operando en los mismos mercados locales.

Los impactos del programa se estiman utilizando un estimador de diferencias en diferencias, con ponderación de probabilidad inversa (IPW, inverse propensity weighting) para balancear las posibles diferencias en características de base entre los PPMVs control y los de la intervención. El uso de la gama completa de las funciones de NaijaCare no conllevó a una mejora significativa en la mayoría de los resultados principales de interés, incluyendo: un índice de prácticas de negocio (−1.4 ± 1.6, *P =* 0.09), el número de medicinas no falsificadas proveídas (0.2 ± 0.4, *P =* 0.25), la percepción de los clientes de la calidad del PPMV (−0.4 ± 0.3, *P =* 0.03), cambios en el número de clientes regulares (−4.0 ± 5.3, *P =* 0.13), y el rol del PPMV durante la pandemia de COVID-19 (por ejemplo, no significativamente más dado a vender una gama de productos de prevención del COVID). Entre los PPMVs participantes, el nivel de participación con la plataforma fue bajo, destacando el involucrar al usuario como un determinante clave para el éxito de una plataforma. El subconjunto de PPMVs que sí usó frecuentemente NaijaCare, mejoró sus prácticas de mantenimiento de registros (0.28 ± 0.27, *P =* 0.05), pero también reportó ganancias diarias significativamente más bajas que las de los PPMVs control (−3759 ± 3417, *P =* 0.04), posiblemente indicando que algunos factores de empuje hacia usar la plataforma, como sería el sufrir dificultades comerciales, fueron responsables de los resultados adversos. Dado que la disponibilidad de la plataforma coincidió con un periodo tumultuoso de cambios traído por la pandemia de COVID-19, estas irrupciones pueden haber tenido un tiempo de intervención y efectividad limitados.

**RESUMO:**

Uma plataforma online—intitulada NaijaCare—ofereceu encomendas digitais, desenvolvimento empresarial, intercâmbio entre pares e formação em competências empresariais a vendedores informais de medicamentos em Lagos, na Nigéria. Os resultados destes vendedores (conhecidos como PPMV—vendedores de medicamentos patenteados e proprietários), que tinham participado numa ronda anterior da programação NaijaCare, são comparados com os de outros PPMV que operam nos mesmos mercados locais. Os impactos do programa são estimados, utilizando um estimador de diferenças em diferenças, com ponderação de propensão inversa para equilibrar possíveis diferenças nas características de base entre PPMV de intervenção e controlo. A gama completa de características da NaijaCare não conduziu a melhorias significativas na maioria dos principais resultados de interesse, incluindo: um índice de práticas comerciais (−1,4 ± 1,6, *P =* 0,09), o número de medicamentos não contrafeitos fornecidos (0,2 ± 0,4, *P =* 0,25), perceção do cliente sobre a qualidade do PPMV (−0,4 ± 0,3, *P =* 0,03), alterações no número de clientes regulares (−4,0 ± 5,3, *P =* 0,13) e papel do PPMV durante a pandemia da COVID-19 (por exemplo, não é significativamente mais provável que venda uma gama de produtos de prevenção da COVID). Entre todos os PPMV participantes, o envolvimento com a plataforma foi baixo, destacando assim o envolvimento como um determinante chave do sucesso da plataforma. O subconjunto de PPMV que utilizavam frequentemente a NaijaCare melhorou as suas práticas de manutenção de registos (0,28 ± 0,27, *P =* 0,05), mas também relatou lucros diários significativamente mais baixos do que os PPMV de controlo (−3759 ± 3417, *P =* 0,04), indicando possivelmente que os fatores de pressão para o envolvimento na plataforma, tais como dificuldades comerciais, foram responsáveis pelos resultados adversos observados. Como a disponibilidade da plataforma coincidiu com um período tumultuoso de mudança provocado pela pandemia da COVID-19, estas perturbações podem ter limitado a utilização e a eficácia da intervenção.

**RÉSUMÉ:**

Une plateforme en ligne – intitulée NaijaCare – proposait des commandes numériques, un développement commercial, des échanges entre pairs et des formations aux compétences commerciales aux vendeurs informels de médicaments à Lagos, au Nigéria. Les résultats de ces vendeurs (connus comme les PPMV, le sigle en anglais de « vendeurs de médicaments brevetés et exclusifs ») qui avaient participé à un cycle antérieur du programme NaijaCare sont comparés à ceux d’autres PPMV opérant sur les mêmes marchés locaux. Les impacts du programme sont estimés à l’aide d’un estimateur de différence des différences, avec une pondération de propension inverse pour équilibrer les différences possibles dans les caractéristiques de base entre les PPMV d’intervention et de contrôle. La gamme complète des fonctions de NaijaCare n’a pas conduit à des améliorations significatives dans la plupart des principaux résultats étudiés, notamment: un indice de pratique commerciale (−1,4 ± 1,6, *P =* 0,09), le nombre d’offre de médicaments non contrefaits (0,2 ± 0,4, *P =* 0,25), la perception des clients de la qualité du PPMV (−0,4 ± 0,3, *P =* 0,03), les changements dans le nombre de clients réguliers (−4,0 ± 5,3, *P =* 0,13) et le rôle du PPMV pendant la pandémie de COVID-19 (par exemple, pas significativement plus susceptible de vendre une gamme de produits de prévention du COVID). Parmi tous les PPMV participants, l’engagement envers la plateforme était faible, soulignant ainsi que l’engagement est un déterminant clé du succès de la plateforme. Le groupe de PPMV qui utilisaient fréquemment NaijaCare ont amélioré leurs pratiques d’enregistrement de données (0,28 ± 0,27, *P =* 0,05), mais ont également signalé des bénéfices quotidiens significativement inférieurs à ceux des PPMV témoins (−3759 ± 3417, *P =* 0,04), ce qui indique peut-être que les facteurs d’incitation à l’engagement à l’utilisation de la plateforme, tels que les difficultés commerciales, étaient responsables des résultats indésirables observés. La disponibilité de la plateforme ayant coïncidé avec une période tumultueuse de changement provoquée par la pandémie de COVID-19, ces perturbations peuvent avoir limité l’utilisation et l’efficacité des interventions.

## INTRODUCTION

The role of private local pharmacies in low- and middle-income countries (LMICs) in contributing to public health initiatives is significant, and interventions to enhance their role have been explored [1]. Digital health technologies (DHTs) have been shown to improve access to noncommunicable disease care in LMICs [2]. However, the lack of a specific regulatory framework for e-pharmacy in LMICs poses challenges, including the sale of prescription-only medicines without a prescription and the sale of substandard and falsified medicines [3]. Despite these challenges, DHTs have the potential to enhance remote health care delivery in pharmaceutical care, with mobile health applications, phone calls and video calls showing promise in improving health-related outcomes [4].

This is also the case in Nigeria, where private pharmacies have increasingly been used, but their effectiveness needs to be enhanced. One of the main challenges in the Nigerian health care system has been the shortage of pharmacies and access to them. A novel set of informal actors emerged to fill the gap—patent and proprietary medicine vendors (PPMVs), who provide pharmaceutical products and could be a source of accurate health advice in the community [5]. A PPMV is defined as ‘a person without formal training in pharmacy who sells orthodox pharmaceutical products on a retail basis for profit’ [6]. PPMVs serve as the first point of acute care for the majority of the Nigerian population and over time, the range of products and services that PPMVs provide has increased; PPMVs sell not only medicine to their clients but also non-health products such as beverages, airtime and food [7].

To regulate this market, PPMVs are required to register with the government as medicine vendors. Despite efforts from the Pharmacists Council of Nigeria to regulate PPMV practices and the types of medicine they can sell, adherence is low; by 2008, only 38% of PPMVs in Lagos were registered with the council. Without registration, the council cannot support the PPMVs, provide training to them or properly regulate the quality and types of medicines that they sell.

PPMVs have been identified as an important source of medicine for childhood illnesses and for adults seeking treatment for malaria in Nigeria [8], yet PPMVs lack access to quality-assured supply chains to stock the products required to treat common illnesses [9]. This gap presented an opportunity to support PPMVs through an online platform where they could order essential medicines and other products that they sell at their stores and receive advice and training to improve their businesses. NaijaCare, a mobile phone–based digital platform that the digital and mobile services provider Every1 Mobile offered, through a grant from Unilever, is one such effort.

The initial NaijaCare platform was designed to provide the NaijaCare Academy, which provided online training on business and medicine practices; a chatroom where PPMVs can discuss business and health topics; a ‘Get Advice’ feature through which pharmacists introduce themselves to PPMVs and provide information; and a knowledge base of online access to articles with answers to the most common questions that PPMVs have and frequently asked questions. NaijaCare’s theory of change suggested that, by offering PPMVs the NaijaCare Academy courses and business peer support through discussion threads and frequently asked questions, the platform could increase knowledge, skills and confidence gained through acquisition of knowledge about business and financial best practices. It was hoped that these steps would increase PPMVs’ sales and profits, in part through increased customer loyalty, and, consequently, improve their livelihoods.

In turn, the platform could also improve population health by improving the quality of service that PPMVs delivered. In particular, the NaijaCare Shop guaranteed that PPMVs were obtaining products from a reliable provider, in this case a verified supplier of over-the-counter medicines. The channels for improving service quality included the PPMVs’ increased knowledge and confidence regarding the concept of quality health care; a reduction in sales of counterfeit medicine; and on the client side, greater access to health care services. Note that regarding the sale of counterfeit medicine, the theory of change assumes that counterfeits enter the supply chain at the producer or supplier level. To the extent that vendors themselves may dilute active ingredients to increase profit per unit of sale, the intervention would not directly address this possible source of low-quality drugs.

The NaijaCare platform was introduced in two phases. Phase 1 was implemented in 2018 and included the four platform features described above. During this phase, 205 PPMVs in Lagos enrolled in NaijaCare; some were also receiving in-person pharmaceutical training through a program called IntegratE. Phase 2 was implemented in February 2020, during which new PPMVs were enrolled (115, all recruited through IntegratE), and additional features were added to the NaijaCare platform. (Further details on the rollout of NaijaCare are available in Annex A within the supplementary materials file).

Unfortunately, additional Phase 2 features were changed because of the COVID-19 pandemic. The features originally intended to be included were the NaijaCare Shop, a voucher scheme for PPMVs to offer vouchers on specific products to their clients to generate customer loyalty, and a primary health care referral feature. COVID-19 rendered intractable both the voucher scheme for clients and referral to primary health care facilities, so the Phase 2 additional features were the NaijaCare Shop and information for PPMVs about COVID-19 prevention methods. To help PPMVs generate business during the COVID-19 pandemic, products to prevent the spread of the virus were included in the NaijaCare Shop.

The objective of this impact evaluation was to determine the impact of Phase 2 of the NaijaCare platform on the businesses of the 165 PPMVs that were part of both Phases 1 and 2 of NaijaCare but not trained in IntegratE, which medicines they provided, and client satisfaction with services delivered.

## STUDY DESIGN AND METHODS

### Evaluation design

The evaluation uses a difference-in-difference methodology that compares changes in outcomes from baseline to follow-up for NaijaCare PPMVs who participated in Phases 1 and 2 and were not trained in IntegratE with those of a set of control PPMVs who did not receive any intervention (neither NaijaCare nor IntegratE). The control group consisted of a random sample of PPMVs operating in the same market as the NaijaCare PPMVs but who did not participate in NaijaCare or IntegratE.

Because program implementation required the newly enrolled Phase 2 PPMVs to receive the IntegratE in-person training program and satisfy certain requirements such as Pharmacists Council of Nigeria certification, the newly enrolled PPMVs were not part of this evaluation, as participation in IntegratE could confound any NaijaCare program impact. Correspondingly, any non-NaijaCare PPMV who was eligible for the control group but participated in IntegratE was excluded from the study to avoid inferential difficulties that may ascribe observed gains to IntegratE rather than NaijaCare. The control group therefore consisted of a random sample of all PPMVs operating in the same market as the NaijaCare PPMVs but did not participate in the IntegratE program.

### Study sample

Of the 165 PPMVs eligible for inclusion in the study, 30 were randomly selected on a geographically stratified basis. Stratification was at the geospatial level of a local market, defined according to a 500-meter radius around a selected PPMV. PPMVs were stratified so that only one PPMV was eligible for selection in each possible nonoverlapping 500-meter radius. Once these local markets were selected all other PPMVs operating in each local market were enumerated and invited to participate in the study (with the exception of any IntegratE PPMV who happened to operate in a selected market). Any additional NaijaCare PPMVs operating in the selected markets were included in the study, along with all non-NaijaCare PPMVs who did not participate in IntegratE. This selection process resulted in a treatment group of 67 NaijaCare PPMVs and 170 comparison PPMVs operating in 30 markets (including the 30 centroidal PPMVs). Geographic information system software (ArcGIS, Esri, Redlands, CA) was used to determine the neighboring NaijaCare PPMVs within a 500-meter radius of one another, and markets were randomly selected using Stata statistical software (Stata Corp, College Station, tX).

### Estimation framework

The main estimation equation is a canonical two-period difference-in-differences specification,


$$ {Y}_{itm}={\gamma}_0+{\gamma}_1{T}_{im}+{\gamma}_2{P}_t+{\gamma}_3{T}_{im}{P}_t+{F}_m+{\varepsilon}_{itm} $$

in which outcome *Y* is indexed according to drug seller *i* in market *m* observed at time *t* (baseline or follow-up). Sellers are indexed according to a binary treatment indicator *T* that takes the value of 1 for NaijaCare PPMVs and 0 for control PPMVs. The follow-up period is indexed according to a binary period indicator *P* that takes the value of 1 for endline and 0 for baseline. The interaction term between *P* and *T*, with associated coefficient ${\gamma}_3$, estimates the impact of program exposure (i.e. the difference-in-differences indicator). To control for time-invariant market-level characteristics, a market fixed effect, *F_m_*, is included. Standard errors are clustered at the market level to account for observational dependence within markets of arbitrary form. Coefficients were estimated using ordinary least squares regression. The identifying assumption in a difference-in-difference framework is known as the parallel trends assumption. This assumption requires that the temporal trends observed among control PPMVs represent the trends that would have occurred among NaijaCare participants if not for the influence of the NaijaCare program.

One possible threat to the parallel trends assumption is that enrollment and participation in the NaijaCare program was voluntary and that program participants might therefore systematically differ from nonparticipants in a variety of characteristics. Therefore, participation propensity scores were estimated on the basis of baseline information and applied to the analysis through inverse propensity weighting to increase the comparability of controls with treated PPMVs [10]. Propensity weights were estimated using information on fixed characteristics that program participation was unlikely to influence. Details of this approach are given in Annex B.

### Data collection

Baseline data collection began in February 2020 and consisted of listing all the PPMV outlets in each selected market. Two hundred thirty-seven PPMVs eligible for the study in the selected markets had a complete or near-complete listing.

Face-to-face interviews with PPMVs and their customers were conducted from February to March 2020. Information was collected on sociodemographic characteristics, outlet features and business practices of the PPMVs before they received full access to NaijaCare Phase 2 features. A sample of up to six medicines was collected from PPMVs to test for quality. (See AnnexC for details on medicine selection and testing.) PPMVs were compensated with ₦500 worth of mobile phone airtime at the end of the interview.

To assess the customer base, including client satisfaction with the products and services received from PPMVs, up to 10 clients of each surveyed PPMV were interviewed using time-location sampling. Eligibility criteria for the client interview included at least 18 years of age and having bought medicine or received a health care–related service from the PPMV. The first 10 clients seeking care in the randomly selected time period were invited for interview. Each client was compensated with ₦300 worth of airtime at the end of the interview.

Although the COVID-19 pandemic, declared in March 2020, did not interrupt the first round of baseline data collection, introduction of coronavirus tools on the NaijaCare platform presented an opportunity to add another study objective. An additional round of phone data collection took place between April and June 2020 during which the same PPMVs were re-interviewed with a short COVID-19-related questionnaire, and 10 clients were interviewed using the original questionnaire plus a new COVID-19 section. For the client interviews, a specific day was randomly assigned to each PPMV, and PPMVs asked clients for their contact information if they were willing to participate in the interview. Given this additional round of data collection, there was a maximum of 20 client baseline interviews per PPMV. Clients interviewed over the phone were compensated with ₦700 worth of airtime. For each successful client interview, PPMVs received ₦300 worth of airtime as an additional incentive for coordinating the client interviews.

Endline data collection was conducted in June and July 2021. In-person endline data were collected from the same 265 PPMVs who consented to interview at baseline, and a maximum of 10 client interviews per PPMV outlet was conducted. Medicines were again sampled from the PPMV outlets, and the questionnaires containing the COVID-19 modules plus a few additional questions were used. (See AnnexC for further information on data collection.) The endline client instrument included the same questions as the baseline questionnaire, the COVID-19 module, and additional client satisfaction questions. An additional phone survey was introduced to collect recontact data on baseline clients for whom phone numbers were collected during the second-round baseline interviews, during which client loyalty, client satisfaction, demand for goods and services and changes in COVID knowledge were measured. At the end of the interviews, PPMVs and clients were compensated with ₦500 worth of airtime as compensation for the time spent participating in the survey.

**Figure 1:**
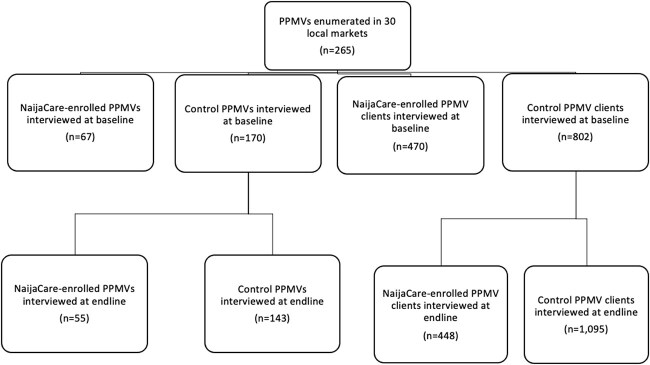
Study design and size. *Note:* PPMV stands for patent and proprietary medicine vendor.

*Note:* PPMV stands fo patent and proprietary medicine vendor.

Figure 1 shows contact and recontact rates for PPMVs and clients at baseline and endline; 39 PPMVs (12 NaijaCare, 27 control) interviewed at baseline were lost to follow-up for a variety of reasons, most commonly business closure. Annex G presents attrition determinant analysis that suggests little risk of bias due to difference in attrition between NaijaCare and control respondents.

### Outcome measures

The evaluation was designed to determine the effects of accessing the full capabilities of NaijaCare for PPMVs who were part of Phase 1 and Phase 2 on five priority outcomes.

The first outcome is related to professional capacity and business capability. A business performance index (BPI) was created to measure whether there was improvement in PPMVs’ professional and business performance through regular use of the practices taught in the courses, peer support and ask-the-expert features that NaijaCare offered. The BPI aggregated individual business practice questions with weights determined by principal components analysis.

The second outcome measured the quality of medicines offered, a general concern in pharmaceutical markets in Nigeria. Around the time of study, up to 30% of hypertensive medicine in Lagos, Nigeria, were falsely labeled [11]; 25% of branded ciprofloxacin tested in Lagos did not contain enough active ingredient to pass the chemical assay test [12]. For this second outcome, up to six specified medicine samples were purchased from study PPMVs and analyzed in a laboratory to determine the rate of counterfeit medicine in circulation for treatment and control PPMVs at baseline and endline. NaijaCare hoped to reduce the amount of counterfeit medicine in circulation in participating PPMV outlets by reducing purchases from unreliable vendors while expanding digital ordering and making quality-assured medicines and fast-moving consumer goods available for PPMVs to provide.

The third outcome involved client satisfaction as a measure of service quality. A client satisfaction index that aggregates across individual satisfaction questions using weights determined by principal components analysis was created to measure whether client perceptions of the quality of care that NaijaCare-enrolled PPMVs provided was better than the care that control PPMVs provided. Dimensions of satisfaction included business improvements that clients noticed and availability of good-quality medicines. Details on outcome index construction are given in AnnexD.

The fourth outcome concerned the possible growth of PPMVs’ client base as a result of a change in service quality. Clients were asked whether their usual source of medicine was from a primary health center, from a NaijaCare PPMV (if surveyed at a participating PPMV), from a control PPMV (if surveyed at a control PPMV) or from a pharmacy. Responses at endline were compared with those from baseline to determine whether participation in NaijaCare resulted in growth in client base, greater client loyalty and better reputation than the rest of the PPMV marketplace. PPMVs also reported the number of clients purchasing drugs in a typical day, which was used as an additional measure of the customer base.

A fifth outcome concerned PPMV knowledge about COVID-19 prevention products and services. This outcome explored PPMVs’ role as information providers and suppliers of goods and services to reduce transmission. After the onset of the pandemic, NaijaCare served as a source of information and of coronavirus prevention products available through online ordering.

An additional objective was to determine the cost of the NaijaCare platform. The results of the cost analysis are discussed below.

## RESULTS

Tables 1 and 2 present the mean baseline characteristics of the PPMV and client samples as well as the normalized mean differences between the NaijaCare and control PPMVs (and their clients). The tables show both the unadjusted means and differences across NaijaCare and control PPMVs and then same measures estimated applying inverse propensity weights. The characteristics included in the table are those that inform the treatment likelihood regression that generates the propensity weights (Annex B).

**Table 1:** Patent and proprietary medicine vendor (PPMV) characteristics at baseline

**Characteristic**	**Unweighted estimations**			**Weighted estimations**		
	**Control**	**Treatment**	**Normalized mean difference**	**Control**	**Treatment**	**Normalized mean difference**
Number of PPMVs in the local market	10.46	10.22	−0.06	10.31	11.38	0.28
Age of PPMV	38.39	41.99	0.35^*^^*^	38.91	37.18	−0.17
Female (proportion)	0.65	0.72	0.13	0.66	0.67	0.00
First language Yoruba	0.56	0.72	0.34^*^	0.58	0.48	−0.20
First language English	0.68	0.76	0.17	0.69	0.60	−0.19
Internet on mobile phone	0.78	0.93	0.36^*^^*^^*^	0.81	0.84	0.07
Ever received health training	0.70	0.93	0.47^*^^*^^*^	0.75	0.81	0.13
Received health training in person	0.69	0.91	0.43^*^^*^^*^	0.74	0.81	0.13
Years working as a PPMV	9.79	11.94	0.27^*^	10.21	10.11	−0.01
Years working as a PPMV in that outlet	6.66	9.51	0.39^*^^*^	7.29	8.08	0.11
Provides diagnostic services at PPMV outlet	0.17	0.33	0.40^*^^*^	0.20	0.32	0.30
Provides medicine that does not require a prescription at PPMV outlet	0.13	0.03	−0.20^*^^*^^*^	0.11	0.18	0.15
Follows waste disposal best practices (is collected or is landfill)	0.88	0.94	0.20	0.90	0.95	0.16
Has medical equipment or uses technology to provide medical services	0.25	0.51	0.54^*^^*^^*^	0.29	0.25	−0.08
Log of income from outlet in preceding calendar month	10.02	10.13	0.09	10.07	10.13	0.05
Log of daily profit amount at endline	7.41	7.59	0.18	7.47	7.52	0.05
Compares prices with other PPMVs prices	0.52	0.67	0.31^*^^*^	0.56	0.67	0.21
Number of workers at outlet	1.58	1.55	−0.03	1.59	1.74	0.16
Time per client (minutes)	5.92	6.91	0.21^*^	6.21	5.86	−0.07
Number of observations	170	67		170	67	

*P* < ^*^.10, ^*^^*^.05, ^*^^*^^*^.01.

**Table 2:** Client characteristics at baseline

**Characteristic**	**Unweighted**			**Weighted**		
	**Control**	**Treatment**	**Normalized mean difference**	**Control**	**Treatment**	**Normalized mean difference**
Age	34.79	35.70	0.08	34.89	35.95	0.09
Gender	0.52	0.54	0.03	0.52	0.58	0.12
Household size	3.95	4.29	0.15^*^^*^	3.94	4.36	0.19^*^^*^^*^
Has secondary education or higher	0.85	0.81	−0.10	0.84	0.81	−0.08
Reason for visit to pharmacist: obtain prescription medicine	0.14	0.19	0.11^*^	0.14	0.16	0.04
Reason for visit to pharmacist: obtain nonprescription medicine	0.87	0.83	−0.09	0.87	0.87	0.01
Availability of medications and appliances needed	0.88	0.92	0.15	0.89	0.93	0.13
Price of medicines	0.97	0.97	0.01	0.97	0.98	0.04
PPMV provides good advice on health problems	0.87	0.93	0.25^*^^*^^*^	0.87	0.91	0.19^*^
Client satisfaction index	−0.09	0.12	0.15^*^^*^	−0.07	0.00	0.05
Confidence that medicine is high quality and manufactured appropriately	0.96	0.99	0.16^*^^*^^*^	0.96	0.99	0.17^*^^*^^*^
Number of observations	802	470		80	470	

*P* < ^*^.10, ^*^^*^.05, ^*^^*^^*^.01.

Because participation in the NaijaCare program is voluntary, NaijaCare participants differ from other PPMVs in notable ways, being, on average, 3.5 years older, somewhat more likely to be female (72% of NaijaCare PPMVs versus 65% of control PPMVs), and more likely to have received formal health training. Given that NaijaCare is a digital platform, it is not surprising that NaijaCare PPMVs were more likely than controls to have Internet access on their mobile phone (93% versus 78%). In terms of PPMV practices, NaijaCare PPMVs were substantially more likely to provide diagnostic services and have medical equipment at the outlet and significantly less likely to report providing medicine without a prescription.

All the indicators mentioned above have a normalized mean difference greater than 0.10 and most greater than 0.20, indicating sufficient characteristic difference that may bias naïve impact estimates [13]. These differences are substantially less after application of inverse propensity weights (Table 1). Few characteristics have a weighted normalized mean difference greater than 0.20, and no difference is significantly different from 0 at standard levels, suggesting that the inverse propensity-weighted difference-in-differences analysis constructs a more suitable PPMV comparison group for NaijaCare users.

Table 2 presents the same baseline characteristic analysis for the client sample, which finds fewer differences at baseline than in the PPMV sample. Examining various characteristics such as age, sex and educational attainment, only average household size was significantly different, with NaijaCare PPMV clients living in slightly larger households (4.3 versus 4.0 household members). NaijaCare clients were also generally happier with services received at baseline, reporting higher satisfaction, greater confidence that medicine received was of high quality, the belief that the PPMV provided good advice, and being more likely to seek medicine with a prescription. These differences may be because of the influence of NaijaCare Phase 1 or because of preexisting differences correlated with the decision to use NaijaCare. Most of these differences, with the exception of general satisfaction with services and seeking medicine with a prescription, persisted after inverse propensity weighting, possibly suggesting that the baseline differences in outcomes may be due to gains from Phase 1 rather than selective uptake of NaijaCare.

Table 3 presents the estimates of program impact for five main outcomes at the outlet level. Columns 1 through 5 present unweighted regressions for the outcomes: business performance (BPI), rate of counterfeit medicine, count of counterfeit medicines sampled, number of clients seeking medicine in a day, and number of regular clients (as reported by the PPMV). Columns 6 through 10 repeat the analysis with the same outcomes now with inverse propensity weights applied to the impact regressions.

**Table 3:** Patent and proprietary medicine vendor (PPMV) results

Variable	Unweighted					Weighted				
	(1)	(2)	(3)	(4)	(5)	(6)	(7)	(8)	(9)	(10)
	Business Performance Index	Rate of counterfeit medicine in circulation	Number of counterfeit medicines sampled per PPMV	Clients looking for medicine in a regular day	Regular customers of the outlet	Business Performance Index	Rate of counterfeit medicine in circulation	Number of counterfeit medicines sampled per PPMV	Clients looking for medicine in a regular day	Regular customers of the outlet
NaijaCare treatment vs control group	0.512	−0.0057	−0.0024	1.219	2.239^*^	0.950^*^	−0.0022	−0.0196	−0.864	2.580
Endline (vs baseline)	0.0217	−0.048^*^^*^^*^	−0.188^*^^*^^*^	1.462^*^^*^	−1.397	0.0505	−0.0436^*^^*^	−0.170^*^^*^	0.816	−2.163^*^^*^
Treatment impact	−0.194	0.0150	0.0555	−3.413^*^^*^	−2.915^*^	−1.373^*^	0.0436	0.243	−0.856	−3.958
Constant	−0.223	0.105^*^^*^^*^	0.426^*^^*^^*^	12.79^*^^*^^*^	13.42^*^^*^^*^	−0.187	0.105^*^^*^^*^	0.424^*^^*^^*^	13.37^*^^*^^*^	13.84^*^^*^^*^
										
Adjusted R-squared	0.009	0.025	0.023	0.008	0.016	0.053	0.016	0.019	0.007	0.051

*Note:* Difference-in-difference estimation of the impact of the program using with robust standard errors clustered at the market level. There are 435 observations in each column. For weighted estimations, propensity score weights were used.

*P* < ^*^.10, ^*^^*^.05, ^*^^*^^*^.01.

No statistically significant difference was found in BPI between treated PPMVs and controls in the unweighted specification (−0.2 ± 0.7, *P =* 0.61). In contrast, the inverse propensity–weighted specification shows a greater decline in the BPI performance indicator for NaijaCare PPMVs than for controls (−1.4 ± 1.6, *P =* 0.09), although NaijaCare PPMVs had a higher mean BPI at baseline, suggesting a return of treated PPMVs to the same level of business practice as controls. There was no discernible impact on the counterfeit medicine measures (number of counterfeit ingredients: 0.2 ± 0.4, *P =* 0.25), although there was a notable secular decline in the rate of counterfeit medicines from baseline to endline, as evidenced by the significant negative coefficient on the period indicator (−0.2 ± 0.1, *P =* 0.02). This suggests a generally improving situation in Lagos regarding prevalence of counterfeit medicines. (Annex E present the results according to individual active ingredients.)

The measures of customer use declined more for NaijaCare PPMVs over time than for control PPMVs, with a decline of 3.0 to 4.0 regular customers, depending on the measure, although these differences were not statistically significant at standard levels in the propensity–weighted impact estimates (unweighted: −3 ± 3.2, *P =* 0.07; weighted: −4 ± 5.2, *P =* 0.13). Annex E also examines the sale of products related to COVID-19 prevention such as face masks and hand sanitizer. NaijaCare PPMVs were no more likely to stock these products than controls, indicating little additional impact of the dedicated COVID-19 curriculum with regard to stocking of preventive commodities, at least over and above the general messaging that was available to all PPMVs. There was also no change in the recommendations given by the PPMVs to clients presenting with covid symptoms.

Table 4 presents program impact for the main outcomes assessed at the client level. In unweighted results, NaijaCare PPMV clients reported a decline in satisfaction from baseline to endline (−0.4 ± 0.3, *P =* 0.04). On the other hand more clients reported switching to NaijaCare PPMVs (0.06 ± 0.05, *P =* 0.04). These two findings remain in the inverse propensity–weighted specifications, and the point estimates are even greater in magnitude, with ~10% of NaijaCare PPMV customers having switched to a NaijaCare PPMV in the previous year (0.10 ± 0.05, *P =* 0.001), and PPMV client satisfaction falling ~0.45 standard deviations, back toward a level more equal to that reported by clients of comparison PPMVs (−0.46 ± 0.29, *P =* 0.03). (At baseline, NaijaCare PPMV clients reported greater satisfaction with service.)

**Table 4:** Client-level outcomes

Variable	Unweighted				Weighted			
	(1)	(2)	(3)	(4)	(5)	(6)	(7)	(8)
	Client satisfaction index	Changed pharmacist in last year	Switched in last 6 months from receiving free medicine to buying it	Switched in last 6 months from buying medicine to receiving it for free	Client satisfaction index	Changed pharmacist in last year	Switched in last 6 months from receiving free medicine to buying it	Switched in last 6 months from buying medicine to receiving it for free
Study treatment group: NaijaCare (vs control)	0.297^*^^*^^*^	−0.0356^*^^*^	0.0107	0.000557	0.300^*^^*^^*^	−0.0393^*^^*^^*^	0.00329	0.00701
Endline (vs baseline)	0.132	−0.000219	0.0940^*^^*^^*^	0.000339	0.136	−0.000929	0.0905^*^^*^^*^	0.000427
Treatment impact	−0.359^*^^*^	0.0555^*^^*^	−0.0494	0.00234	−0.457^*^^*^^*^	0.102^*^^*^^*^	−0.0347	−0.00467
Constant	−0.112	0.0677^*^^*^^*^	0.0214^*^^*^^*^	0.00386^*^	−0.0807	0.0668^*^^*^^*^	0.0195^*^^*^^*^	0.00325^*^
								
R-squared	0.007	0.004	0.027	0.000	0.014	0.020	0.026	0.001

*P* < ^*^.10, ^*^^*^.05, ^*^^*^^*^.01.

There are 2815 observations that informs the analysis in each column.

The digital nature of NaijaCare necessitates that users engage with the platform for it to provide the intended benefits. In light of this, additional analysis was done to investigate possible differences in impacts according to frequency of platform usage. Data from the implementation agency show that, from January 2020 to July 2021, ~20% of Phase 1 NaijaCare users used the platform at least once per month. This rate of usage was suggested by implementers as a minimum metric of regular engagement. (Analysis of usage data is reported in Annex F.) Because PPMVs in Phase 1 had had access to NaijaCare since 2018, new Phase 2 material and features were created to maintain user engagement and to re-engage PPMVs who had disengaged from the platform. Since overall use did not appreciably increase, this goal was not fully met.

Nevertheless, if the analysis restricts the set of treated PPMVs to active users of the NaijaCare platform at baseline, defined as PPMVs who self-reported use of NaijaCare more than once per month, the results are somewhat different. Frequent NaijaCare users improved their record keeping (0.28 ± 0.27, *P =* 0.05) but these frequent users also reported a significantly greater decline in daily profits than controls (−3759 ± 3417, *P =* 0.04).Given possible endogeneity in the decision to use the platform with respect to unobservable characteristics, it is difficult to interpret theses associations as causal, but these findings are consistent with a push factor for increased usage—PPMVs experiencing business difficulties seek solutions, which can include the content and services that NaijaCare offers.

Alongside estimates of impact, the study also estimated implementation and ongoing costs associated with the NaijaCare program, differentiating between costs incurred by a funder and users of the system and accounting for both financial and economic costs. The approach aligns with recently developed guidance for the economic evaluation of digital health interventions tailored to their attributes and conceptualization of value (see [14]). Cost data is often a neglected component of evaluative efforts, however these data can provide useful insights for understanding the cost dynamics of NaijaCare and may also be a useful input for future economic evaluation and decision analytic modeling efforts.

A critical aspect of costing a digital health intervention is the difference between development and implementation costs. Digital health interventions typically have high development and set-up costs, with decreasing marginal costs over time, especially given increasing numbers of participating units. Indeed, marginal costs at scale may very well be negligible. Development costs are related to the initial conceptualization and design of the intervention and are expected to be irrecoverable but possibly transferable to other settings and contexts. Implementation costs are also irrecoverable but are associated with the one-time direct provision of the intervention in the particular context. Recurring costs are ongoing costs required per use or user of the intervention, and are associated with the marginal costs of the intervention per user. In a complex intervention like NaijaCare that incorporates elements of adaptive design while expanding its user base, it is not possible to differentiate between development, implementation, and recurring cost because the costs are incurred from a similar source. For this analysis, estimates were made in consultation with the provider based on existing expenditures.

Estimates of the implementation and recurring costs, as informed by costing and program data from the provider (Every1 Mobile), were differentiated according to funder and users (PPMVs) where applicable. Because there was some overlap in time and actions of staff in implementation and recurring activities, author judgement was required in discussion with program staff to assign appropriate allocations.

Table 5 details the implementation costs associated with the NaijaCare program, assigned to appropriate categories based on functionality. Because there was no initiation fee or other financial cost, time costs to PPMV operators were estimated based on reported average set-up time and local market wages.

**Table 5:** Implementation costs of NaijaCare program: funders and patent and proprietary medicine vendor (PPMV) operators

Cost perspective	Cost description	Total value (USD; estimate if required)	Duration
Funder	Personnel expenses related to planning (ideation and planning)	83 564	Oct 17-Sep 20
	Personnel expenses related to set-up	186 121	Oct 17-Sep 18
	Attributable costs of software development and systems design, including costs of original platform and enhancements	687 550	Oct 19-Sep 20
	Attributable costs of online shop, including license fee for external software, subsidies and set-up costs	104 498	Apr 18-Sep 18
	Costs of onboarding and PPMV training	20 620	Jan 18-Mar 18
	Personnel expenses related to planning and set-up features that have since been retired	230 969	Oct 17-Sep 20
	*Total*	*694 527*	
PPMV operators	Time PPMVs spent during training and onboarding sessions on system use training	8.89 per PPMV	Initial use per PPMV operator
	Cost to initiate program	*0*	

The total in-country implementation cost for NaijaCare is estimated to be US$694527 (measured in 2020 US dollars), which incorporates a cost field related to retired features of the program that were considered but discontinued. Adaptation to the constraints and feasibility of the local context is a critical element of implementation, and although these costs do not have any direct attributable impact on the current functionality of the program, they are an important element of local implementation. Future funding models for the program may include use charges to users to cover other implementation costs. PPMVs also incur one-time implementation costs in terms of time spent learning about the functionality and use of the platform. This is estimated to be 3 h and is calculated as ~₦3375 (US$8.89) per user. Although this is negligible in the context of total implementation costs, the impact of this cost at scale is expected to be significant.

The ongoing costs of the NaijaCare program that providers and PPMVs incur are detailed in Table 6. The recurring costs are low in relation to the set-up costs, as is common with digital interventions that require high upfront investment. As with implementation costs, PPMVs incur limited costs that are largely driven by time on site. The low data requirements of the site and Nigeria’s relatively low cost of mobile data also minimize recurring costs. The recurring cost estimates indicate that, at scale, the intervention would require only a modest impact to be cost-effective and that there may be a range of feasible cost-recovery options for a sustainable financial model to be developed.

**Table 6:** Recurring costs: funder and patent and proprietary medicine vendors (PPMVs) per month

Responsible party	Cost description	Cost element	Cost (US$)
Funder	Personnel in the field and running the platform	Local staff	8595
		Travel and overhead	3586
		Monthly expenses to run platform	538
	Platform	Maintenance (e.g. software program support, excluding routine personnel costs above)	1603
	Medicine supply system	Monthly operational payments to providers	1049
		*Total*	*15 371*
PPMVs	Time using platform: Average time on site	Hours monetized using price of labor estimate	6.48 (₦2691)
	Data costs to access platform	Cost/megabyte	0.002–0.006 (₦0.79–2.60)
		*Total*	6.49 (₦2693)

## DISCUSSION AND CONCLUSION

This impact evaluation of the NaijaCare platform did not find a significant impact of Phase 2 of the NaijaCare program on most of the outcomes of interest. This finding also applies to frequent users of the platform, who did report a positive change in business practices but alongside a greater decline in daily profits than controls (perhaps due to COVID-19-related disruptions). Given the nonexperimental design of this evaluation, various explanations of these results may be valid, including the possibility that business troubles spurred PPMVs to look for solutions through engagement with NaijaCare and related options.

Study limitations included the influence of several unanticipated changes that affected program implementation and, potentially, study results. These changers included the COVID-19 pandemic and subsequent disruptions to social and economic life, implementer changes to intervention features, and overall declines in counterfeit medicine distribution. Regarding the disruptions experienced over the pandemic period, PPMVs were asked at endline if COVID-19 had affected sales at their outlets. 74% of control PPMVs and 77% of treatment PPMVs responded that profits had decreased because of the pandemic, attesting to the turbulent period during which this study was conducted.

The extended exposure of the treatment population to NaijaCare (since 2018) might also have affected the study results. High-frequency users (~20% of the study population) were defined as those accessing the application at least once a month (therefore even fewer users were accessing NaijaCare more than once a month). This level of engagement would barely register as frequent use, especially for a mobile phone app that had a chat feature, references and resource materials, a shop from which to order new inventory and other engagement tools. One conclusion is that widespread low use of the NaijaCare app likely contributed to the (lack of) study results.

Engagement is critical to the success of a new intervention. The low level of use of the NaijaCare platform suggests that that intervention never really became of central relevance to PPMVs. It’s possible that a more complete intervention over a much shorter duration may generate greater engagement among PPMVs. PPMVs who were busy and doing relatively well may have paid less attention to this intervention, while PPMVs whose businesses were not doing well may have hoped to turn things around, in part, through this intervention. The issue of uptake of an intervention has important implications when measuring the effectiveness of an intervention and needs attention in future research.

Regarding determinants of uptake, the NaijaCare Theory of Change proposed a range of intervention elements including business mentoring, business education, credit, a customer loyalty scheme, diagnostic services, professional education, professional mentoring and referrals [15]. Yet, very few of these design elements actually became part of the intervention. Perhaps, a less complex set of expectations and fewer design elements would have had a higher likelihood of getting implemented, particularly in the complex environment of Nigeria. With a new digital intervention, a simpler design may have greater likelihood of success. Several of these elements, including referrals or diagnostic services or credit seem ambitious for a project with a relatively short intervention period.

Instead, while a series of new business behaviors were anticipated from PPMVs, the drivers of these new behaviors were platform components which continued to be added slowly over time. NaijaCare remained in a design phase in which different intervention elements were added slowly. For example, an eCommerce component was not part of the original project design. It was an add-on that remained in a pilot phase with a minority of PPMVs using the eCommerce sporadically. This approach to intervention design with intervention elements being added periodically and perhaps without sufficient intensity may have resulted in low interest and engagement of PPMVs.

From a broader perspective, the offer of a new technology is not necessarily sufficient to drive key behavioral changes and health outcomes. As users are ultimately the ones to decide whether to engage with a new technology, this user experience needs to be foremost in mind when constructing a theory of change for an emergent health related technology. With these lessons in mind, subsequent efforts to assist informal medicine vendors may prove more successful—the recurring cost estimates indicate that, at scale, this intervention would have required only modest impact to be cost effective.

## Supplementary Material

Nigeria-Supplementary_Material_oqae035

## Data Availability

Study data will be available, upon request and in de-identified format, from the World Bank’s data portal, data.worldbank.org.
