# Supplementary material for: Evaluation of the effects of a digital health platform on business and medical practices of informal medicine vendors in Lagos, Nigeria
Source: Oxf Open Digit Health. 2024 Dec 2;2(Suppl 2):ii56–65. doi: 10.1093/oodh/oqae035 (PMC11936323; doi:10.1093/oodh/oqae035)
Supplement: Nigeria-Supplementary_Material_oqae035 [file Nigeria-Supplementary_Material_oqae035.pdf]

Supplementary Materials:

Evaluation of the Effects of a Digital Health Platform on Business and Medical Practices of Informal Medicine Vendors in Lagos, Nigeria

Laura A. Ruiz-Gaona[1,] Jed Friedman[2], Nejma Cheikh[3], Thomas Wilkinson[3], Mengxiao Wang [3], Jasmine Vicencio[3], Sohail Agha[4], Marelize Gorgens[3]

1. J-PAL, Health, Cambridge, MA 02142
2. Development Research Group, World Bank, Washington DC 20433
3. Health and Nutrition Global Practice, World Bank, Washington DC 20433
4. Global Health Visions, Saugerties, NY 12477

<u>*Corresponding Author</u>:
Marelize Gorgens
mgorgens@worldbank.org
HNP Global Practice, World Bank
1818 H Street NW,
Washington, DC 20433

Technical annexes for “Evaluation of the Effects of a Digital Health Platform on Business and Medical Practices of Informal Medicine Vendors in Lagos, Nigeria”

Annex A.	Program Description and Theory of Change
Annex B.	Propensity Score Weighting
Annex C.	Data Collection
Annex D.	Index Outcomes of Interest
Annex E.	Other Results, Pharmaceuticals and COVID-19 Prevention
Annex F.	NaijaCare Usage Data and Results by Usage
Annex G.	Attrition Analysis
Annex H.	Cost Analysis

Annex A. Program Description and Theory of Change

NaijaCare is a mobile phone–based digital platform that the digital and mobile services provider Every1 Mobile created and provided through a grant from Unilever. Initial workshops with patent and proprietary medicine vendors (PPMVs) in Nigeria to determine their needs revealed that PPMVs saw themselves not only as medicine and health care providers, but also as business owners and, as such, were interested in improving their business plans, stock control, and client loyalty.

NaijaCare was introduced in two phases. Phase 1 started in 2018 and consisted of enrolling 205 PPMVs in Lagos, 40 of whom were also working with IntegratE—an initiative led by the Society for Family Health that provided the PPMVs with in-person training on topics such as quality of family planning services. NaijaCare's features include education and mentoring through the NaijaCare academy, which offers eight courses in business and health; a chat room updated weekly where PPMVs can discuss business and health topics; and a pharmacist mentorship available through the "Get Advice" feature, where community pharmacists introduce themselves, Expert articles based on questions that the PPMVs most commonly asked and frequently asked questions were posted. During Phase 1, some PPMVs also participated in a pilot program for ordering medicine and fast-moving consumer goods through NaijaCare's online ordering platform: NaijaCare Shop. PPMVs supply not only medicine to clients, but also non-health care products, including beverages, airtime, and food.

NaijaCare Phase 2 started in February 2020, and during this phase, 115 new PPMVs, recruited through IntegratE, joined NaijaCare. Phase 2 offered new and existing PPMVs on the platform the same features as Phase 1, with the addition of an online ordering tool available for all PPMVs in the program. During this phase, some PPMVs also participated in a pilot program to receive

credit for online orders. Initially, Phase 2 was planned to include a scheme to enable participating PPMVs to provide vouchers for specific products to their clients to generate loyalty and a hospital referral feature that would allow PPMVs to refer their clients to hospitals if needed while simultaneously alerting hospitals to expect those individuals. Unfortunately implementation challenges prevented the two features from being introduced. Instead, because of the COVID-19 pandemic, a feature was added that provided information to PPMVs about COVID-19 prevention methods, and products to prevent the spread of the virus were included in the stock of goods available through NaijaCare Shop.

To enroll PPMVs in the platform during Phase 1, Every1 Mobile approached PPMVs who attended meetings organized by the National Association of Patent and Proprietary Medicines, an association of PPMVs in Lagos, where they meet to share important messages, news, and information regarding their businesses. The PPMVs were presented with the NaijaCare program and could enroll if they had an Internet-enabled mobile phone. Every1 Mobile provided PPMVs with information about the advantages of using NaijaCare through in-person visits, workshops, and one-on-one WhatsApp chats.

Enrollment follow a different procedure in Phase 2; PPMVs who had been in face-to-face trainings provided by IntegratE were encouraged to enroll in NaijaCare.

NaijaCare’s theory of change suggested that, by offering PPMVs the NaijaCare Academy courses and business peer support through discussion threads and frequently asked questions, the platform could increase knowledge, skills, and confidence gained through acquisition of knowledge about business and financial best practices and client loyalty. It was hoped that these steps would increase PPMVs’ sales and profits and, consequently, improve their livelihoods.

In turn, the health care delivery courses offered through the NaijaCare Academy, peer support through chat and frequently asked questions, the Get Advice feature, and the NaijaCare Shop could improve population health by improving the quality of service that PPMVs delivered. In particular, the NaijaCare Shop guaranteed that PPMVs were obtaining products from a reliable provider, in this case DrugStoc, a verified supplier of over-the-counter medicines. The channels for improving service quality included the PPMVs' increased knowledge and confidence regarding the concept of quality health care; a reduction in sales of counterfeit medicine; and on the client side, a reduction in the cost of health care products and greater access to health care services.

Annex B. Propensity Score Weighting

Using data from before and after the NaijaCare intervention, this impact evaluation adopted a difference-in-differences identification strategy that could be complemented with inverse propensity weights to better balance baseline characteristics across treatment and control patent and proprietary medicine vendors (PPMVs). Because PPMVs could self-enroll into the program, conditional on having access to a mobile phone with Internet access, treatment PPMVs may have systematically differed from controls in characteristics that can, at least, partially determine the identified priority outcomes.

The difference-in-differences methodology covers two time periods, $t = \{1,2\}$, with the baseline period defined at $t = 1$, and accounts for unobserved characteristics affecting Phase 2 program up-take that are fixed over time, conditional on the identifying assumption of parallel trends. To control for observable characteristics of the PPMVs that might differ between the treatment and control groups, the propensity score–weighting methodology captures the influence of covariates on program participation in a single score that is used to create a weight for each PPMV (treatment and controls) that represents their probability of participating in the program.

The first step in creating inverse propensity weights is selecting the covariates—observable characteristics at baseline that may jointly affect participation and outcomes of interest. Selected characteristics include:

- Age
- Gender
- First language Yoruba
- First language English
- Access to a mobile phone
- Internet on mobile phone
- Ever received medical training
- Received health training in person
- Years working as PPMV
- Years working as PPMV in that specific outlet

- Services provided at PPMV: diagnostics
- Services provided at PPMV: injections
- Waste disposal best practice: collected or landfill
- Have medical equipment or use any technology to provide medical services
- Log of baseline income
- Log of baseline daily profit amount
- Compared prices with those of other PPMVs
- Number of workers at the outlet
- Time per client
- Quartile 1 of market density: number of PPMVs in the cluster
- Quartile 2 of market density: number of PPMVs in the cluster
- Quartile 3 of market density: number of PPMVs in the cluster

After selecting the covariates, the program participation score is calculated for each PPMV using a *Probit* model as follows,

$$T_i = \gamma_0 + \gamma_1 X_i + \varepsilon_{itm}$$

where the outcome T represents the binary variable treatment that takes a value of 1 for treatment PPMVs and 0 for control PPMVs. i indexes the individual PPMV. The characteristic vector X contains all the covariates previously selected and is associated with the coefficient vector γ_1. Standard errors are clustered at the market level, *m*. Using this equation, the probability of participating in the program for each PPMV can be predicted.

There is a region of common support in the propensity score between the treatment and control groups where the distributions of the propensity score for both groups overlap (Figure B1).

Figure B1. Distribution of propensity scores

A Distribution of propensity scores stratified by treatment B. Propensity scores, pooled

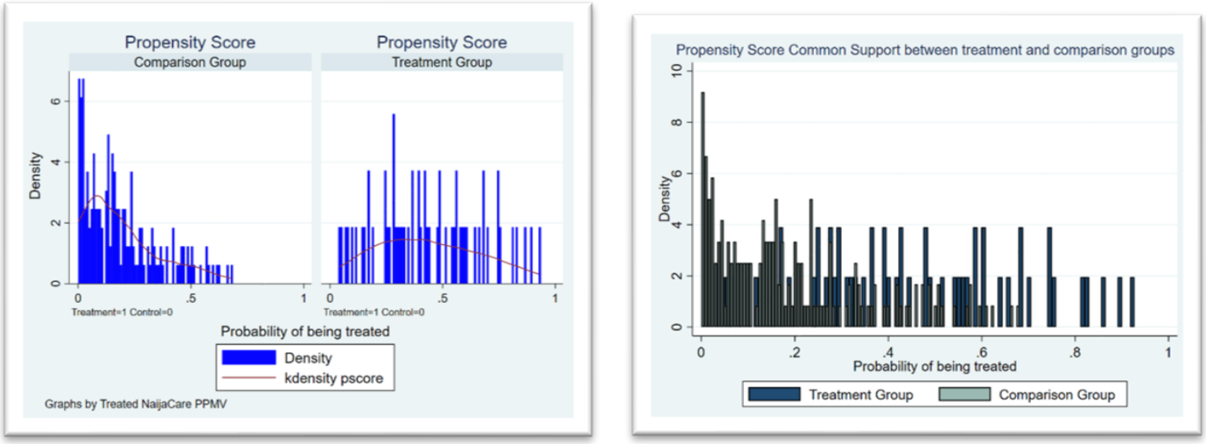

The region of common support between the groups is between 0.042 and 0.93. Since this commons support region is so broad, all PPMVs are included in the main analysis. (If comparison PPMVs with a propensity score below 0.042 are dropped, impact results are largely the same as reported in the main part of the report.) The last step is to create propensity weights for each PPMV based on predicted propensity scores using the formula

$$w^T = \frac{1}{propensity\ score}\ if\ Treatment = 1$$

for treatment participants and

$$w^C = \frac{1}{(1 - propensity\ score)}\ if\ Treatment = 0$$

for controls.

Annex C. Data Collection

The listing exercise revealed considerable variety in the number of patent and proprietary medicine vendors (PPMVs) per market, with some markets having as few as two competitors per treated PPMV and other markets with as many as 17. Given this heterogeneity in market density, the research team decided to select all non-NaijaCare non-IntegratE PPMVs in each local market for interview, so all non-NaijaCare and non-IntegratE PPMVs in the local markets were defined as the control group.

Two hundred ninety-six PPMVs and pharmacies were listed within the preselected local markets; 63 refused to be interviewed, withdrew their consent, or did not complete the interview.

Two hundred fifty-nine PPMVs with a completed listing met the requirements to continue in the study, and six NaijaCare PPMVs with incomplete listings were included in the study as well. Therefore, the final PPMV sample included 265 PPMV outlets in the 30 randomly selected markets.

The research team partnered with the local data collection agency Hanovia Limited, which had also conducted the listing exercise, to conduct baseline data collection in February and March 2020.

Five prespecified medicines of interest were sampled in all PPMVs (treatment and control), including an antimalarial (artemether/lumefantrine; Clartem); an antihelminth (albendazole; Tanzol tablets); a corticosteroid (prednisone); a nonsteroidal antiinflammatory drug (diclofenac); and a short-acting, selective beta2-adrenergic receptor agonist (salbutamol; Asmalyn). The specific brands mentioned in the parentheses were offered to NaijaCare PPMVs through the platform's online ordering tool. The medicine with the highest volume of sales for each PPMV in

addition to the five mentioned above was also sampled for analysis. If the PPMV did not have any of the prespecified medicines of interest in stock, up to three medicines with the greatest volume of sales were sampled as replacements, conditional on them being in tablet or liquid forms. A maximum of six medicines could be purchased (i.e. sampled) from each PPMV. The sampled medicines were labeled and sent to a laboratory to be tested for the validity of active ingredients.

For client interviews, time-location sampling was conducted by dividing PPMV outlet operation hours into three shifts (morning: 7:00–13:00; afternoon: 13:00–17:00; evening: 17:00–22:00). For the follow-up round of baseline data collection, the PPMVs asked the first 20 clients who met the client interview requirements outlined above and who went to the PPMV on a specific day that was randomly assigned to them if they wanted to give their contact information for an interview. If so, the data collection agency contacted them over the phone to respond to the survey. This time, there was randomization of the day but not the time. Given this additional round of data collection, there were a maximum of 20 client baseline interviews per PPMV instead of 10. PPMVs and clients were compensated at the end of each interview with ₦700 worth of airtime. For each successful client interview, the PPMV also received ₦300 worth of airtime as an additional incentive for coordinating the interview.

At endline, the PPMV survey instrument included the original PPMV questionnaire, the COVID-19 module with some additional questions, and a new module on entrepreneurship self-efficacy. The medicines sampled included the same five medicines of interest as those sampled at baseline, the medicine with the highest volume of sales sampled at baseline, and the medicine with the highest volume of sales at endline if it was different from those already sampled. All medicines had to be in tablet or liquid form. If the enumerator could not sample up to seven medicines

according to the above-mentioned criteria, the following medicines were to be sampled in this order—paracetamol, thiamine, amoxicillin, mebendazole, diphenhydramine hydrochloride, ibuprofen—until seven medicines were sampled. The medicines were sent to the same lab as at baseline.

For the endline client survey, data collection was randomized from Monday to Saturday, excluding Thursday morning, which is when PPMVs are usually closed to sanitize their premises and therefore unavailable to assist with client interviews, and only two times were considered for randomization (morning: 7:00–14:00; afternoon: 13:00–20:00). The reasons for this change were that baseline data collection showed that PPMV outlets were closed on some of the randomized days and the time shifts were too short to complete the desired number of client interviews. At the end of the interviews, clients were compensated with ₦500 worth of airtime incentives as compensation for time spent participating in the survey.

Annex D. Index Outcomes of Interest

The business performance index was created using measures of best practices that were taught to patent and proprietary medicine vendors (PPMVs) through the online courses, including the measures below. Each measure was converted to a binary yes/no variable and aggregated with weights from a principal components analysis.

- PPMV asks clients for illness history.
- Medicines and supplies are stored and organized according to expiration date.
- Records are kept of medicine purchased for selling purposes.
- Prices are fixed so that the PPMV does not bargain with the client.
- PPMV has a stock-keeping system.
- PPMV keeps track of medicines' expiration dates.
- Medicines are kept separate from other products.
- Medicines that have expired are kept separate from the rest of the medicines.
- PPMV has ever created a business plan.
- PPMV keeps a record of products bought and sold.
- PPMV has a written budget,
- PPMV keeps regular records of work.
- PPMV keeps track of earnings each month.
- PPMV keeps record of products and quantities in stock.
- Business and personal cash are kept separate.
- Records are kept of possible risks and consequences for the business
- PPMV uses tools to keep track of stock in outlet or shop.

For the rate of counterfeit medicine, the research team generated an estimate for each PPMV that aggregates across the counterfeit binary measure for the five predetermined medicines of interest using the formula in the equation below.

$$\frac{Number\ of\ counterfeit\ medicines\ found\ in\ the\ PPMV\ outlet}{Total\ medicines\ sampled\ in\ the\ PPMV\ outlet}$$

For the client satisfaction index, the scores that PPMV clients gave regarding their satisfaction with the following items were aggregated using principal component analysis.

- Outlet cleanliness
- Comfort and convenience of waiting areas
- Availability of medications and appliances needed
- Price of medicines

- Advice provided on health problems
- Information provided on family planning products and services
- Information provided on health centers
- Rewards provided for frequent purchases

Annex E. Other Results, Pharmaceuticals and COVID-19 Prevention

Table E.1 presents the results for the main sampled medicines. Analysis of the five medicines of interest, disaggregating the results for each medicine, shows a statistically significant reduction over time of the number of counterfeit albendazole, diclofenac, and salbutamol samples. The results are consistent when using propensity score weights. There was an increase in the number of counterfeit artemether/lumefantrine samples, but no statistically significant difference between the treatment and control groups can be attributed to the NaijaCare program for any of these medicines.

For products that PPMVs sold that can be associated with COVID-19 prevention and that were offered to PPMVs through the NaijaCare platform, none of the coefficients showed a statistically significant impact, with the exception of the unweighted regression for hand sanitizer (Table F.2).

Table E.1. Rates of Counterfeit Medicines

Variable	Albendazole	Albendazole (weighted)	Diclofenac	Diclofenac (weighted)	Prednisolone	Prednisolone (weighted)	Salbutamol	Salbutamol (weighted)	Artemether/lumefantrine	Artemether/lumefantrine (weighted)
Treatment PPMV	0.0265 (0.0612)	-0.131 (0.0962)	-0.0548 (0.0487)	-0.0404 (0.0533)	-0.0319 (0.0367)	-0.0461 (0.0301)	0.0696 (0.0974)	0.0122 (0.141)	0 (7.60e-10)	0 (2.09e-09)
Endline	-0.0809** (0.0402)	-0.157 (0.0958)	-0.140*** (0.0311)	-0.121*** (0.0283)	-0.0405 (0.0278)	-0.0436 (0.0345)	-0.197*** (0.0541)	-0.164** (0.0663)	0.119*** (0.0282)	0.120*** (0.0353)
Program impact	-0.0645 (0.0684)	0.0928 (0.102)	0.0397 (0.0498)	0.0272 (0.0540)	0.0215 (0.0461)	0.0239 (0.0418)	-0.0320 (0.113)	0.132 (0.246)	0.0806 (0.0611)	0.127 (0.127)
Constant	0.140*** (0.0338)	0.210** (0.0866)	0.155*** (0.0292)	0.135*** (0.0248)	0.0735*** (0.0225)	0.0795*** (0.0238)	0.253*** (0.0481)	0.225*** (0.0598)	0 (4.39e-10)	-0
Number of observations	326	314	396	382	349	341	235	230	389	374
R-squared	0.031	0.063	0.060	0.052	0.009	0.015	0.081	0.034	0.089	0.126

P<***.01, **.05, *.10.

Table E.2. COVID-19 Prevention Products Sold Through Patent and Proprietary Medicine Vendor Outlets

Variable	Unweighted							Weighted						
	Hand sanitizer	Face masks	Rubbing alcoho	Latex gloves	Antibacterial wipes	Anti-microbial	None	Hand sanitizer	Face masks	Rubbing alcoho	Latex gloves	Antibacterial wipes	Anti-microbial	None

			l			spray or liquid				l			spray or liquid	
NaijaCare treatment	-0.0371	-0.120	0.0933	-0.00945	0.0296	-0.0900*	0.0127	-0.0486	-0.0481	-0.0446	-0.0739	-0.0233	-0.150***	-0.00491
Endline (vs baseline)	-0.0565	0.0458	-0.114	-0.114**	0.0139	0.0264	0.0542	-0.0538	0.0581	-0.104	-0.127**	0.00114	0.00719	0.0514
Program treatment	0.140*	0.151	-0.0355	0.0566	0.0846	0.0342	-0.0133	0.0288	-0.0465	-0.0212	-0.0132	0.148	0.0926	0.178
Constant	0.754***	0.687***	0.657***	0.776***	0.254***	0.157***	0.0373*	0.743***	0.672***	0.656***	0.781***	0.269***	0.172***	0.0406
R-squared	0.007	0.018	0.023	0.013	0.009	0.012	0.010	0.003	0.008	0.016	0.027	0.017	0.040	0.090

Note: Difference-in-differences estimation of the impact of the program. For weighted estimations, propensity score weights were used. There are 391 observations in the analysis.
P<***.01, **.05, *.10.

Annex F. NaijaCare Usage Data and Results by Usage

Usage data from the implementing firm showed that approximately 20 percent of the 174 patent and proprietary medicine vendors (PPMVs) who registered on the platform since the start of Phase 1 were frequent users of the platform—accessing the platform at least once per month in all months (Table F.1).

Table F.1. NaijaCare Users

Phase 1 active NaijaCare users	Jan-20	Feb-20	Mar-20	Apr-20	May-20	Jun-20	Jul-20	Aug-20	Sep-20	Oct-20	Nov-20	Dec-20	Jan-21	Feb-21	Mar-21	Apr-21	May-21	Jun-21	Jul-21
Number	39	41	45	46	54	45	45	32	36	40	50	34	40	35	40	39	36	31	28
Percentage of total users	22.4	23.6	25.9	26.4	31.0	25.9	25.9	18.4	20.7	23.0	28.7	19.5	23.0	20.1	23.0	22.4	20.7	17.8	16.1

Note: There were 174 total users.

	Number
Changed from frequent to infrequent user	7
Stayed an infrequent user	10

Stayed a frequent user	12
Changed from infrequent to frequent user	1
Total	30

Information was also collected on changes in usage data from the PPMV respondent survey (response rates for this question was low – only 30 treated PPMVs provided an answer). Participants who answered that they were registered in NaijaCare and who were confirmed in the treatment group were asked about their use of the platform. Most did not change their usage from baseline to endline, although seven became infrequent users, and one became a frequent user (Table F.2).

Table F.2. Change in Use from Baseline to Endline

If impact analysis is restricted only to frequent users of NaijaCare, and assessed against the full set of comparator PPMVs, it is apparent that frequent users exhibit some improvement in certain individual business practices over the study period but also experience a decline in profits. As stated in the main text, these associations do not necessarily represent a causal relation with respect to program involvement, but may suggest a push factor in so far as PPMVs encountering difficulties (reduced profits) actively seek solutions, including those offered by digital platforms.

Table F.3. Program impact on select indicators, only frequent NaijaCare users

Dependent variable	Record keeping of products and quantities	Keep expiration records	Daily profits
NaijaCare treatment	-0.1405	-0.3472***	2066.3
Endline (vs baseline)	-0.0101	0.0524	1972.6***

Program treatment	0.2767**	0.4619**	-3759.2**
Constant	0.4617***	0.5056***	2619.1***
Adjusted R-squared	0.032	0.120	0.027
Observations	377	247	301

Annex G. Attrition Analysis

Attrition analysis showed that propensity to leave the study before endline data collection was unrelated to treatment status, suggesting little risk of bias from differential attrition. Table G.1 regresses the attrition indicator of a patent and proprietary medicine vendor (PPMV) on a range of baseline characteristics, a treatment indicator, and a treatment indicator interacted with each characteristic.

Table G.1. Correlates of Attrition

Variable	Partial likelihood of attrition
Treatment group	-0.561
Number of PPMVs in local market	-0.00188
Age of PPMV	0.00662*
Sex	-0.0548
First language Yoruba	0.0524
First language English	0.0377
Has access to mobile phone with Internet	0.0119
Has medical training	-0.181
Medical training was in person	0.0685
Time as PPMV	-0.0125**
Time as PPMV in that specific outlet	0.00821
PPMV services: diagnostic	-0.0855
PPMV services: provide medicine that does not require prescription	0.237**
Follows waste disposal best practices (is collected or is landfill)	0.00694
Has medical equipment or uses any technology to provide medical services	-0.0203
Log of monthly income	-0.0421
Log of daily profit amount	0.0106
Compares prices with other PPMVs’ prices	-0.0253
Number of workers at the outlet	-0.00470
Time per client	-0.00579
Treatment group*number of PPMVs in local market	0.00330
Treatment group*age of PPMV	0.00970
Treatment group*sex	-0.0340
Treatment group*first language: Yoruba	-0.0513
Treatment group*first language: English	-0.0720
Treatment group*has access to mobile phone with Internet	-0.178
Treatment group*has medical training	-0.355
Treatment group*medical training was in person	0.116
Treatment group*time as PPMV	0.00413
Treatment group*time as PPMV in that specific outlet	-0.0111

Treatment group*PPMV services: diagnostic	0.101
Treatment group*PPMV services: provide medicine that does not require prescription	0.228
Treatment group*waste disposal best practice (is collected or is landfill)	-0.134
Treatment group*have medical equipment or use any technology to provide medical services	0.00838
Treatment group*log of monthly income	0.107
Treatment group*log of daily profit amount	-0.0177
Treatment group*compare prices toother PPMVs prices	0.105
Treatment group*number of workers at the outlet	-0.0804
Treatment group*time per client	-0.00836
Constant	0.425
Number of observations	237
R-squared	0.179

Note: PPMV, patent and proprietary medicine vendor. The table reports the result of a linear probability model. The independent variable is a binary indicator indicating whether or not the baseline PPMV attrited by endline. Standard errors clustered at the market level. There are 30 markets.

Annex H. Cost Analysis

The cost analysis estimated implementation and ongoing costs associated with the NaijaCare program, differentiating between costs incurred by a funder and users of the system and accounting for financial and economic costs. The approach aligns with recently developed guidance from the World Bank detailing a framework for the economic evaluation of digital health interventions tailored to their attributes and conceptualization of value (see Wilkinson et al., 2023). The digital health intervention framework consists of a series of steps and analytical principles to improve the information that economic evaluation of digital health interventions generates and can be applied to a range of analytical requirements from preliminary analysis to comprehensive economic evaluations. Given the challenges of establishing a causal pathway between intervention and outcomes that would traditionally be incorporated into a health economic evaluation, this analysis focused on the costing elements of the framework. These data will provide useful insights for immediate understanding of the cost dynamics of NaijaCare and may also be a useful input for future economic evaluation and decision analytic modeling efforts.

An important element of the analysis is consideration of how factors that may influence the economic impact of an intervention differ between digital and nondigital interventions. The broad aim of the NaijaCare platform is to improve health care and the financial standing of informal shop owners, and there are numerous examples of nondigital programs for patent and proprietary medicine vendors (PPMVs) in Nigeria with comparable aims that invariably rely on personnel for aspects such as training and quality improvement support. The role of scale is critical in any economic assessment, because digital health interventions typically have high development and set-up costs, with decreasing marginal costs over time and increasing numbers of participating units, as well as depending on implementation structure. Marginal costs at scale are potentially negligible.

Understanding of the intended decision maker or user of the information that the analysis generates informs how the perspective for costing is determined. Philanthropic institutions (Unilever; Bill and Melinda Gates Foundation) funded NaijaCare with an intended long-term vision that the program would be self-sufficient, with platform users generating funding. Although the program may have indirect expenditure implications for the Nigerian public health service through changes in demand and supply of primary health care services, the local or federal Nigerian government or health department is not a direct investor in the program, so expenditures on this program are not expected to have a direct opportunity cost with regard to money available for public health.

A critical aspect of the representation of costs in the economic evaluation of a digital health intervention is the difference between development and implementation, as well as recurring (or ongoing) costs. Development costs are related to the initial conceptualization and design of the intervention and are expected to be irrecoverable but transferable to other settings and contexts. Implementation costs are also irrecoverable but are associated with the one-time direct provision of the intervention in the particular context. Recurring costs are ongoing, are costs required per use or user of the intervention, and are associated with the marginal costs of the intervention per user. In a complex intervention like NaijaCare that incorporates elements of adaptive design and is developing new functionality while expanding its user base, it is not possible to differentiate between development, implementation, and recurring cost because the costs are incurred from a similar source. For this analysis, estimates were made in consultation with the provider based on existing expenditures; subsequent data collection may facilitate representation and allocation of cost structures.

Estimates of the implementation and recurring costs, as informed by costing and program data from the provider (Every1 Mobile), were differentiated according to funder and users (PPMVs) where applicable. Because there was some overlap in time and actions of staff in implementation and recurring activities, author judgement was required in discussion with program staff to assign appropriate allocations.

Table H.1 details the implementation costs associated with the NaijaCare program, assigned to appropriate categories based on functionality and approach to cost accounting. Because there was no initiation fee or other financial cost, time costs to PPMV operators were estimated based on reported average set-up time and local market wages.

Table H.1: Implementation Costs of NaijaCare Program: Funders and Patent and Proprietary Medicine Vendor (PPMV) Operators

Cost perspective	Cost description	Total value (USD; estimate if required)	Duration
Funder	Personnel expenses related to planning (ideation and planning)	83,564	Oct 17-Sep 20
	Personnel expenses related to set-up	186,121	Oct 17-Sep 18
	Attributable costs of software development and systems design, including costs of original platform and enhancements	68,7550	Oct 19-Sep 20
	Attributable costs of online shop, including license fee for external software, subsidies, and set-up costs	104,498	Apr 18-Sep 18
	Costs of onboarding and PPMV training	20,620	Jan 18-Mar 18
	Personnel expenses related to planning and set-up features that have since been retired	230,969	Oct 17-Sep 20
	Total	*694,527*	
PPMV operators	Time PPMVs spent during training and onboarding sessions on system use training	8.89 per PPMV	Initial use per PPMV operator
	Cost to initiate program	*0*	

The total in-country implementation cost for NaijaCare is estimated to be US$694,527, which incorporates a cost field related to retired features of the program that were considered but discontinued. Adaptation to the constraints and feasibility of the local context is a critical element of implementation, and although these costs do not have any direct attributable impact on the current functionality of the program, they are an important element of local implementation. PPMVs also incur one-time implementation costs in terms of time spent learning about the functionality and use of the platform. This is estimated to be 3 hours and is calculated as approximately ₦3,375 (US$8.89) per user. Although this is negligible in the context of wider implementation costs, the impact of this cost at scale is expected to be significant, and future funding models for the program may include charges to users to cover other implementation costs.

The ongoing costs of the NaijaCare program that providers and PPMVs incur are detailed in Table H.2. The recurring costs are low in relation to the set-up costs, as is common with digital interventions that require high upfront investment. As with implementation costs, intervention users (PPMVs) incur limited costs which are largely driven by time on site. The low data requirements of the site and Nigeria's relatively low cost of mobile data also minimize recurring costs. The recurring cost estimates indicate that, at scale, the intervention would require only a modest impact to be cost-effective and that there may be a range of feasible cost-recovery options for a sustainable financial model to be developed.

Table H.2. Recurring Costs: Funder and Patent and Proprietary Medicine Vendors (PPMVs) per Month

Responsible party	Cost description	Cost element	Cost (US$)
Funder	Personnel in the field and running the platform	Local staff	8,595
		Travel and overhead	3,586

		Monthly expenses to run platform	538
	Platform	Maintenance (e.g., software program support, excluding routine personnel costs above)	1,603
	Medicine supply system	Monthly operational payments to providers	1,049
		Total	*15,371*
PPMVs	Time using platform: Average time on site	Hours monetized using price of labor estimate	6.48 (₦2,691)
	Data costs to access platform	Cost/megabyte	0.002–0.006 (₦0.79–2.60)
		Total	6.49 (₦2,693)
